# Evaluation of a spot-on imidacloprid-moxidectin formulation (Advocate®) for the treatment of naturally occurring esophageal spirocercosis in dogs: a double-blinded, placebo-controlled study

**DOI:** 10.1186/s13071-018-2731-x

**Published:** 2018-03-05

**Authors:** Gilad Segev, Alicia Rojas, Eran Lavy, Marganit Yaffe, Itamar Aroch, Gad Baneth

**Affiliations:** Koret School of Veterinary Medicine, The Hebrew University of Jerusalem, P.O. Box 12, 7610001 Rehovot, Israel

**Keywords:** Canine, *Spirocerca lupi*, Esophagus, Doramectin, Endoscopy, Coproscopy

## Abstract

**Background:**

Dogs are the definitive hosts of *Spirocerca lupi*. Spirocercosis is treated by prolonged avermectin administration by injection or daily oral doses. In this prospective, double-blinded, placebo-controlled, clinical trial, the efficacy of imidacloprid and moxidectin spot-on formulation (Advocate®) was compared to injectable doramectin (Dectomax®). Dogs diagnosed with benign esophageal spirocercosis were divided randomly into doramectin (400 μg/kg IM) or moxidectin and imidacloprid spot-on (2.5–6.25 mg/kg and 10–25 mg/kg, respectively) groups and treated weekly for 12 consecutive weeks. Dogs were followed for 20 weeks by physical examination, owners’ questionnaire, blood work, fecal floatation, PCR and endoscopy.

**Results:**

All the doramectin group dogs (*n* = 10) completed the treatment and follow-up, and the disease had completely resolved in all by week 12. Of the Advocate® group (*n* = 10), four had complete resolution at week 12, four had partial resolution, one dog did not respond to treatment, and one dog was switched to the doramectin protocol on week 5 due to persistent severe clinical signs. PCR analysis was more sensitive in detecting *S. lupi* eggs compared to fecal floatation. Discrepancies were detected on 22 occasions, of which on 20 occasions, the PCR was positive while fecal floatation was negative, and only on two occasions the PCR results were negative while fecal flotation was positive.

**Conclusions:**

The present results indicate that weekly Advocate® spot-on administration may be effective for treating benign esophageal spirocercosis, but is less effective than the currently used injectable doramectin therapy at the dose and duration used herein.

## Background

*Spirocerca lupi* infects several different canid species, which are its definitive hosts, including domestic dogs. Spirocercosis occurs mostly in tropical and subtropical climate areas, and has been emerging in Israel since the 1990s [1–6].

Dogs become infected by ingesting infected intermediate coprophagous beetle hosts or preying on infected paratenic hosts (e.g. birds, lizards and rodents) [5, 7–10]. After ingestion, infective L3 larvae are freed from the intermediate or paratenic host in the dog’s stomach, penetrating the gastric wall, migrating into walls of small gastric arteries, the gastric and celiac arteries and the caudal thoracic aorta, and finally to the caudal thoracic esophageal wall. During migration, L3 larvae mature to the L4 and L5 stages and finally into adults, in the esophageal wall, where they reproduce. The female worm burrows an opening through the esophageal wall, forming a nipple-like structure opening into the esophageal lumen, through which eggs are shed and passed in the feces. The prepatent period lasts 3–6 months. The presence of adult worms in the esophageal wall induces formation of one to several fibrous tissue nodules in a single host [5, 7–10]. Neoplastic transformation of the esophageal nodules to sarcoma occurs in 8–26% of the cases [11, 12].

The clinical signs of spirocercosis often include regurgitation and vomiting, due to esophageal obstruction by the nodules, esophagitis and gastric reflux [5, 13]. Other manifestations of the disease include acute death (due to aortic aneurism rupture), dyspnea and tachypnea (due to aspiration pneumonia, pyothorax, or pulmonary metastases), neurological spinal cord signs (due to aberrant spinal cord migration), lameness (due to arthritis, spondylitis or hypertrophic osteopathy secondary to pulmonary metastases when neoplastic transformation occurs) and thromboembolism [14–21]. Chronic infections also lead to inappetance, weight loss and weakness [22–24].

Antemortem diagnosis of benign esophageal spirocercosis mostly depends on esophagoscopy and coproscopy. The endoscopic diagnosis is based on documenting a caudal esophageal smooth nodule with a ‘nipple-like’ protuberance [2]. Conversely, neoplastic masses associated with spirocercosis are larger and irregular, with hemorrhagic and necrotic surface areas. Previous studies report complete agreement between the diagnosis of neoplasia based on gross endoscopic findings of large superficially necrotic, ulcerative and hemorrhaging esophageal masses and diagnosis based on representative tissue samples obtained for histopathology [23].

Benign esophageal spirocercosis can be treated successfully with doramectin or ivermectin (400 μg/kg SC every 1–2 weeks for 6–12 weeks, or by daily oral therapy at the same dose) or with milbemycin-oxime (11.5 mg on days 0, 7, 28 and then monthly) [13, 24–26]. Conversely, malignant esophageal spirocercosis requires surgical excision, possibly with chemotherapy, and has a poor prognosis [17, 24, 27]. Therefore, benign esophageal spirocercosis needs to be identified and treated early, before neoplastic transformation has occurred, to achieve a successful outcome.

Successful treatment of benign esophageal spirocercosis requires prolonged avermectin therapy by weekly injections or by daily oral avermectin doses or repeated oral milbemycin-oxime doses [13, 24–26]. Such prolonged injectable or oral therapy protocols might decrease owners’ compliance, potentially resulting in treatment failure. Furthermore, the use of doramectin in dogs for spirocercosis is extra-label, and certain dog breeds may be susceptible to avermectin toxicity [28, 29]. Therefore, there is an obvious advantage in finding alternative effective, preferably approved, drugs for treatment of canine spirocercosis, with alternative drug administration routes, to decrease treatment cost, increase owners’ compliance and minimize drug-associated toxicity.

Imidacloprid 10% and moxidectin 2.5% spot-on combination for dogs (Advocate®, Bayer) was previously evaluated for prevention of naturally occurring spirocercosis and for treating experimentally-induced spirocercosis in dogs [30]. In the latter study, 24 experimentally infected dogs were allocated to three groups: (1) untreated controls; (2) prevention group: dogs treated 28 days prior to infection (day 0), at day 0 and then monthly, until day 280 (i.e. 12 treatments); and (3) treatment group: infected dogs treated weekly, starting at day 170 for 19 consecutive weeks [30]. All dogs underwent endoscopy, and were eventually necropsied 308 or 310 days from infection. In that study, Advocate® was effective for prevention and treatment of *S. lupi* infection, although some dogs in treatment group 3 developed esophageal nodules. In a controlled study evaluating Advocate® for prevention of naturally occurring *S. lupi* infection, the drug was administered monthly for nine months to young dogs naturally exposed to *S. lupi*, and has led to a significantly decreased infection rate in the treatment group (1.7%) compared to the control group (35.2%) [31].

We hypothesized that Advocate®, administered topically weekly for 12 weeks will be as effective and safe as weekly doramectin injections (400 μg/kg SC) for 12 weeks in eliminating clinical signs, fecal egg shedding and the benign esophageal nodules in dogs naturally infected with *S. lupi*. The specific aims of this study were to assess the efficacy of Advocate® in dogs with naturally occurring benign esophageal spirocercosis in general, and specifically: (i) assess its efficacy in eliminating clinical signs of benign esophageal spirocercosis compared to doramectin treatment; (ii) evaluate its efficacy in eliminating spirocercal egg shedding compared to doramectin treatment; (iii) assess its efficacy in inducing regression and resolution of *S. lupi*-induced esophageal benign nodules compared to doramectin treatment; and (iv) assess the potential side effects of weekly Advocate^®^ treatment compared to those of the conventional doramectin treatment.

## Methods

### Design

This study was a prospective, double-blinded, placebo-controlled, observational clinical trial conducted at the Hebrew University Veterinary Teaching Hospital (HUVTH). It included dogs diagnosed with benign esophageal spirocercosis with their owners’ signed consent. Dogs were included only if the esophagoscopic morphology of the esophageal nodule was typical of a benign *S. lupi*-induced mass (i.e. smooth, non-ulcerated esophageal masses, with a ‘nipple-like’ protuberance) and there was no evidence of neoplasia, based on esophagoscopy and survey thoracic radiography [32]. Cases suspected of esophageal neoplasia, based on esophagoscopy (i.e. large, ulcerated, bleeding, irregular esophageal wall lesions) [32] and thoracic survey radiography (i.e. large size of a caudal mediastinal mass, or presence of nodule calcification, pulmonary metastases and hypertrophic osteopathy) [2, 23], dogs aged < 6 months, and dogs treated with ivermectin, doramectin, selamectin, moxidectin or milbemycin within 3 months prior to presentation were excluded.

At presentation to the HUVTH, dogs underwent a complete physical examination, blood was obtained for a complete blood count (CBC) and routine serum chemistry, thoracic survey radiography (left and right lateral and ventrodorsal views), upper gastrointestinal endoscopy, routine coproscopy (i.e. direct fecal smear and saturated sugar solution-based fecal flotation for detecting and quantifying *S. lupi* eggs) [33], and polymerase chain reaction (PCR) fecal testing using *S. lupi*-specific primers [34].

The dogs were enrolled consecutively, and were blindly (i.e. the attending clinicians and owners were unaware of the type of treatment) and randomly (by opening sealed envelopes deposited with a single technician prior to the recruitment of dogs) assigned into a doramectin (Dectomax, Zoetis, San Paulo) treatment (control) group (administered at 400 μg/kg, SC q7d for 12 consecutive weeks) or Advocate® treatment (study) group (moxidectin and imidacloprid at 2.5–6.25 mg/kg and 10–25 mg/kg, respectively, spot-on, q7d for 12 consecutive weeks). Dogs were followed for 20 weeks. Dogs allocated to the study group were administered Advocate® spot-on solution and sterile propylene-glycol solution injections (placebo; q7d SC). The control dogs received doramectin injections and a sterile propylene-glycol spot-on (placebo; q7d). The medications used in both treatment groups were provided using identical external packaging, and administered to all dogs by a single technician.

### Follow-up and additional testing

Follow-ups were prescheduled (Table 1). Fecal samples for sugar solution floatation and *S. lupi* PCR were stored at -80 °C pending analysis. The number, size and location of esophageal nodules were recorded, and photos were taken during endoscopy. A questionnaire was completed by the owners at each recheck (Table 2).

**Table 1:** Follow up examinations in dogs diagnosed with benign esophageal spirocercosis enrolled in the study

Week	1	2	3	4	5	6	7	8	9	10	11	12	16	20
Day	0	7	14	21	28	35	42	49	56	63	70	77	105	133
Physical examination	×	×		×		×		×				×	×	×
Treatment	×	×	×	×	×	×	×	×	×	×	×	×		
Complete blood count	×													×
Sample for PCR (frozen)	×	×	×	×		×		×				×	×	×
Serum chemistry	×													×
Fecal floatation	×	×	×	×		×		×				×	×	×
Fecal HRM real-time PCR	×	×	×	×		×		×				×	×	×
Endoscopy	×											×		×
Thoracic survey radiography	×							×				×		×
Owner questionnaire	×		×		×			×				×	×	×

*Abbreviations*: *HRM* high resolution melt; PCR, polymerase chain reaction

Dogs failing to respond to treatment within 4 weeks, based on the attending clinician’s judgment (i.e. no clinical improvement based on the intensity of clinical signs or occurrence of clinical signs deemed adverse drug reactions) underwent an additional upper gastrointestinal endoscopy, and the attending clinicians were made aware of the type of treatment provided, and when the dog had been treated with Advocate®, the option to switch therapy to the conventional doramectin treatment for the rest of the study period was considered.

### DNA extraction and molecular analysis

DNA was extracted from 0.2 g of fecal sample using the Qiagen Fast DNA Stool Mini Kit (Qiagen, Hilden, Germany) with some modifications [34]. DNA purity and concentration were verified using a NanoDrop spectrophotometer (Thermo-Fisher Scientific, Waltham, MA, USA).

After DNA extraction, all samples were tested in triplicates by quantitative PCR, coupled with a high-resolution melt analysis (HRM qPCR) that detects a 135 bp fragment of the internal transcribed spacer 1 (ITS1) of *S. lupi*. Primers SlITS1-F and SlITS1-R were used at final concentrations of 500 mM, and the PCR program was run as previously described [34]. Each PCR run included DNA from a *S. lupi* eggs suspension as positive control, DNA from a *S. lupi*-negative fecal sample as negative control (determined negative by fecal flotation of three consecutive fecal samples) and a non-template control with PCR-grade water (Biological Industries, Beit-Haemek, Israel). Samples were considered positive when all three replicates had been amplified.

Amplicons from positive DNA samples were purified (Exo-SAP, New England Bio-Labs, Ipswich, MA, USA) and sequenced using the BigDye Terminator cycle sequencing chemistry (Applied Biosystems ABI3700 DNA Analyzer and ABI’s Data collection and Sequence analysis software, ABI, Carlsbad, CA, USA).

### Statistical analysis

The distribution pattern of continuous variables was assessed using the Shapiro-Wilk test. Continuous parameters were compared between groups using the Student’s t-test or Mann-Whitney U-test, depending on data distribution. The Friedman test was used to compare changes in continuous variables over > 2 time-points. Comparison of continuous variables of two different time-points was done using the Wilcoxon signed-rank test. The proportions of dichotomous (categorical) variables were compared between groups using Fisher’s exact test. All tests were 2-tailed, and a *P* < 0.05 was considered significant. Statistical analyses were made using a statistical software package (SPSS 22.0, IBM, Chicago, IL, USA).

## Results

Twenty dogs were enrolled in this study, including 13 males (7 castrated) and 7 females (4 spayed), with an overall median age of 72 months (range 12–160 months), and an overall median body weight of 22 kg (range 5–41 kg). There were no significant age and body weight group differences between the study and the control group (Mann-Whitney U-test: *U*_(40)_ = 41.0, *Z* = -0.682, *P* = 0.529 and Mann-Whitney U-test: *U*_(40)_ = 33.0, *Z* = 1.85, *P* = 0.2, respectively). There was no significant difference in body weight between presentation and the primary endpoint (week 12) in both treatment groups. Dogs were of the following breeds: mixed-breed (9 dogs), Labrador retriever, Jack Russell Terrier, English Pointer (2 dogs each) and seven other breeds (1 dog each).

### Clinical signs and owners’ questionnaire

Based on the owners’ questionnaire and laboratory blood work, no adverse reactions were documented to the administration of either doramectin or Advocate® at any of the time points. There were no statistically significant differences in the median scores of any of the answers to the questionnaire questions at presentation, with exception of the general attitude question, for which the median score was lower (i.e. worse) in the control compared to the study group (Mann-Whitney U-test: *U*_(20)_ = 23.5, *Z* = -2.163, *P* = 0.031) (Table 2). The following relates to the change in the questionnaire-generated scores from presentation to the primary endpoint (Week 12). The general attitude score significantly increased (i.e. has improved) in the doramectin group but not in the Advocate® group (Friedman test, *χ*^2^ = 14.622, *df* = 4, *P* = 0.006, Friedman test, *χ*^2^ = 5.772, *df* = 4, *P* = 0.217, respectively) (Table 2). The activity score had also significantly increased in the doramectin group but not in the Advocate® group (Friedman test, *χ*^2^ = 21.463, *df* = 4, *P* < 0.001, Friedman test, *χ*^2^ = 4.873, *df* = 4, *P* = 0.298, respectively) (Table 2). The regurgitation score decreased in the Advocate® group but this decrease was insignificant (Friedman test, *χ*^2^ = 9.128, *df* = 4, *P* = 0.058) (Table 2), while in the doramectin group the decrease was significant (Friedman test, *χ*^2^ = 10.232, *df* = 4, *P* = 0.037) (Table 2). There was no significant change in the salivation score in the Advocate® and the doramectin groups between these two time-points (Friedman test, *χ*^2^ = 0.187, *df* = 4, *P* = 0.996 and Friedman test, *χ*^2^ = 8.694, *df* = 4, *P* = 0.069, respectively) (Table 2), nor was there a significant change in the appetite score between these two time-points in both groups (Friedman test, *χ*^2^ = 0.855, *df* = 4, *P* = 0.931 and Friedman test, *χ*^2^ = 2.028, *df* = 4, *P* = 0.731, respectively) (Table 2).

**Table 2:** Change in owners’ clinical score between presentation and 12 weeks

	Attitude^a^		Activity^b^		Regurgitation^c^		Salivation^d^		Appetite^e^	
	Week 1	Week 12	Week 1	Week 12	Week 1	Week 12	Week 1	Week 12	Week 1	Week 12
Doramectin	5 (3–5)	5 (3–5)	4 (2–5)	5 (2–5)	1.5 (1–4)	1 (1–2)	1 (1–5)	1 (1–5)	5 (1–5)	5 (2–5)
Advocate	3 (1–5)	5 (2–5)	2.5 (1–5)	5 (2–5)	2.5 (1–5)	1 (1–3)	1 (1–5)	1 (1–5)	5 (2–5)	5 (4–5)

^a^1, very depressed, 5 excellent

^b^1, normal; 5, severely decreased

^c^1, none; 5, severe

^d^1, none; 5, severe

^e^1, anorectic; 5, excellent

### CBC and serum chemistry

The CBC and serum chemistry results at presentation and throughout the follow-up period were unremarkable in all dogs, with no significant differences between time-points and between groups at each time-point.

### Survey radiography and endoscopy findings at presentation

A caudal mediastinal mass was identified by survey radiography in all but two dogs (one in each treatment group). In the lateral projection, the mean mass length were 23.6 mm (SD 31.4) and 44.3 mm (SD 28.5) and the mean mass heights were 39 mm (SD 17.1) and 40.3 (SD 21.1) in the Advocate® and doramectin groups, respectively, with no significant differences between groups (t-test: *t*_(20)_ = -1.541, *P* = 0.141 and *t*_(20)_ = -0.142, *P* = 0.889, respectively).

Typical *S. lupi* esophageal nodules were identified on endoscopy in all dogs. The median overall number of nodules identified was 3 (range, 1–7), with no difference between the Advocate® and the doramectin groups (median 2.5, range 1–4 *vs* median 3, range 1–7, respectively; Mann-Whitney U-test: *U*_(20)_ = 35.0, *Z* = -1.168, *P* = 0.243).

### Response to treatment

All the doramectin group dogs had completed the treatment and follow-up protocol, and in all, complete resolution of the esophageal nodules, as noted upon endoscopy, was documented at week 12 from treatment initiation. One dog in the Advocate® group was switched to the doramectin protocol at week 5 due to persistent severe clinical signs. Four of the nine Advocate® group dogs which had completed the 12-week treatment protocol showed complete resolution of the esophageal nodules, as noted upon endoscopy, while in the remaining five, the esophageal nodules did not resolve. Of these latter five, in one, the esophageal nodules’ size remained unchanged, in three, it had decreased by approximately 50% and in one it had decreased by approximately 75%. All these latter five dogs were switched to conventional doramectin therapy, and follow-up repeat endoscopy (week 20) showed complete resolution of the esophageal nodules. In the dog that had been switched from the Advocate® to the doramectin treatment at week 5, complete endoscopic resolution was noted at week 12.

There was no significant difference in the esophageal mass length, as measures on lateral projection of thoracic radiography, between the time of diagnosis and week 12 in the Advocate® group (18.1 ± 27.8 mm *vs* 22.6 ± 35.0 mm; paired sample t-test: *t*_(9)_ = -0.506, *P* = 0.626), but this difference was significant in the doramectin group (44.3 ± 28.5 mm *vs* 4.5 ± 14.6 mm; paired sample t-test: *t*_(10)_ = 4.442, *P* = 0.002). There was a significant decrease in mass height, as evaluated by dorsoventral projection of thoracic radiography, between these two time points in both the Advocate® (36.3 ± 15.7 mm *vs* 20.5 ± 20.0 mm; paired sample t-test: *t*_(9)_ = 2.654, *P* = 0.029) and the doramectin (40.3 ± 21.1 *vs* 14.0 ± 18.7; paired sample t-test: *t*_(10)_ = 5.929, *P* < 0.001) groups.

### Coproscopy results: fecal floatation and HRM qPCR results

In five and six rechecks, due to technical problems, samples were unavailable for fecal floatation and HRM qPCR analysis, respectively. Fecal *S. lupi* eggs were detected at presentation (week 1) by floatation in three and two dogs in the Advocate® and the doramectin groups, respectively, while HRM qPCR results were positive in six and three dogs, respectively (Table 3). Positive fecal floatation results or positive HRM qPCR were detected at presentation and during the follow-up period in 10/10 and in 5/10 dogs in the Advocate® and doramectin groups, respectively.

**Table 3:** Presence of fecal *S. lupi* eggs and DNA based on sugar flotation and high-resolution melt real-time polymerase chain reaction, respectively, in dogs with naturally occurring esophageal spirocercosis treated with Advocate® or doramectin. Dogs number 2, 3, 5, 6, 7 were switched from Advocate® to doramectin treatment on week 12 and dog number 9 was switched from Advocate® to doramectin treatment on week 5

Week/ Dog ID	1	2	3	4	6	8	12	16	20
	FF/PCR	FF/PCR	FF/PCR	FF/PCR	FF/PCR	FF/PCR	FF/PCR	FF/PCR	FF/PCR
Advocate® (treatment) group									
1	+/na	+/+	-/+	+/+	-/-	-/-	-/-	-/+	-/-
2	+/+	+/+	+/+	+/+	+/+	+/+	+/+	-/+	-/-
3	-/+	+/-	-/+	-/+	-/-	-/-	-/-	-/-	-/-
4	+/+	-/-	-/-	-/-	-/-	-/-	-/na	-/-	-/-
5	-/+	-/-	+/-	-/-	-/-	-/-	-/-	-/-	-/-
6	-/-	-/+	-/-	-/-	-/-	-/-	-/-	-/-	-/-
7	-/-	-/+	-/-	+/+	+/+	+/+	+/+	-/+	na/-
8	-/-	-/-	-/+	-/-	-/-	-/-	-/-	-/-	-/-
9	-/+	+/+	-/+	-/+	-/+	-/-	-/-	-/-	na/-
10	-/+	-/+	+/+	-/+	-/-	-/-	-/-	na/-	-/-
Doramectin (control) group									
11	+/na	-/+	-/-	-/+	-/-	-/-	-/-	-/-	-/-
12	-/-	-/-	-/-	-/-	-/-	-/-	-/-	-/-	-/-
13	-/-	-/-	-/-	-/-	-/-	-/-	-/-	-/-	-/-
14	-/-	-/-	-/-	-/-	-/-	-/-	-/-	-/-	-/-
15	na/+	-/-	-/-	-/-	-/-	-/-	-/-	-/-	-/-
16	+/+	-/-	-/-	-/-	-/-	-/-	-/-	-/-	-/-
17	-/-	-/+	-/+	-/+	-/-	-/-	-/-	-/-	-/-
18	na/+	+/+	-/-	+/na	-/-	-/-	-/-	-/-	-/-
19	-/-	-/-	-/-	-/-	-/-	-/-	-/-	-/-	-/-
20	-/na	-/-	-/-	-/-	-/-	-/-	-/-	-/-	na/-

*Abbreviations*: *FF* sugar fecal floatation, *PCR* fecal polymerase chain reaction; *na* not available

Discrepancies between the fecal floatation and the HRM qPCR results were detected on 22 occasions, of which on 20, the HRM qPCR was positive, while fecal floatation was negative, and on two, the HRM qPCR results were negative while fecal flotation was positive. All samples that were positive upon HRM qPCR had DNA sequences that were 100% identical to *S. lupi* GenBank accession number MF425539.

## Discussion

The active ingredients in Advocate® include imidacloprid 10% and moxidectin 2.5%. Evidence suggests that Advocate® is effective for treatment of experimental *S. lupi* infection if administered weekly for 19 consecutive weeks and for prevention of spirocercosis [30, 31]. However, this preparation has never been assessed in a double-blinded placebo-controlled fashion for treating dogs naturally infected with *S. lupi*, and its efficacy has not been directly compared with the conventional avermectin treatment. The results of this study indicate that topical weekly administration of Advocate® (moxidectin 2.5–6.25 mg/kg) for 12 weeks may be considered for treating naturally occurring benign esophageal spirocercosis in dogs, although, at the dose used herein and the duration of 12 weeks, it is less effective compared to the currently used doramectin therapy.

*Spirocerca lupi* infection may be fatal if undiagnosed during its benign stage, due to the risk of neoplastic transformation of the esophageal nodules [11, 23, 32, 35, 36]. Due to the wide use of avermectins, concerns have been raised regarding emergence of resistance to these drugs [37–42]. In Israel, routine preventative treatment is recommended by most veterinarians every three months; however, it is possible that this treatment has decreased efficacy over time due to emerging resistance [3].

In the present study, weekly Advocate® or doramectin treatments were not associated with any notable adverse clinical reactions or laboratory abnormalities, based on the owners’ responses and the blood tests, respectively. Regular topical Advocate® administration is easier for pet owners’ compared to weekly doramectin injections or its prolonged daily oral doramectin or ivermectin administration, which will potentially improve the owners’ compliance. Additionally, Advocate® has been approved for dogs, while the use of ivermectin and doramectin for spirocercosis in dogs is extra-label.

There were no statistically significant group differences in the median owner questionnaire-based scores at presentation, excluding the general attitude question, for which the median score was lower (worse) in the doramectin group compared to the Advocate® group, indicating that the two study groups were comparable at presentation. Some differences were noted, however, in these median scores during the treatment period. Notably, there was a significant improvement over time in the attitude and activity scores in the doramectin group, but not in the Advocate® group, and while the regurgitation score decreased significantly over time in both groups, in the Advocate® group the decrease was insignificant, suggesting that doramectin is more effective compared to Advocate® in eliminating benign esophageal spirocercosis-associated clinical signs at the doses and treatment duration used in this study. When quick clinical response is desired, based on the present results, doramectin should be considered superior to Advocate®.

Complete resolution of the esophageal nodules was recorded in 40% of the Advocate® group dogs, while in the additional 40%, the nodules’ size had decreased by 50–75%, and in one, there was no response at the primary end-point (week 12). In the remaining dog of this group, Advocate® treatment was discontinued due to persistent, non-improving clinical signs. Conversely, all the doramectin group dogs had completely responded to treatment at week 12. All the Advocate® group dogs in which the esophageal nodules had not resolved at week 12, and in the one where the clinical signs failed to improve at week 5 were switched to conventional doramectin treatment, which led to complete endoscopic resolution of the nodules later. Based on these results, doramectin seems to be more effective in eliminating esophageal *S. lupi* nodules compared to Advocate®. However, as 40% of the Advocate® group did show complete resolution of the esophageal nodules, and an additional 40% showed partial resolution at week 12, Advocate® does show efficacy for treating *S. lupi* infection. Possibly, a longer Advocate® treatment period would have led to complete resolution of the esophageal nodules and the clinical signs in a higher proportion of the Advocate® group dogs.

This difference in efficacy between the two treatment groups was also reflected by the presence of *S. lupi* eggs in the feces. While fecal eggs were not detected from day 7 post-initiation of treatment onwards in any doramectin group dog, in the Advocate® group, fecal *S. lupi* eggs were detected in some dogs after the treatment had been initiated, and in two, eggs were persistently detected throughout the study period up to week 12. These results are further supported by the fecal HRM qPCR results, in which 43% (22/51) and 9% (5/56) of the fecal samples collected from dogs during Advocate® and doramectin therapy, respectively, were positive for *S. lupi* DNA.

This study has also demonstrated the low sensitivity of conventional fecal floatation for diagnosing benign esophageal spirocercosis compared to fecal PCR testing, as previously described [3, 5]. In 20/22 cases where discrepancies were noted between these two methods, positive results were obtained by HRM real-time qPCR, while contrary results were noted only in two samples, with positive fecal flotation and negative HRM qPCR. The latter result possibly resulted from presence of fecal PCR inhibitors, incomplete fecal sample homogenization for DNA extraction or misidentification of eggs by microscopy.

This study had several limitations. First, the size of both treatment groups was limited. Secondly, as these dogs had naturally occurring clinical spirocercosis, variability existed between groups. However, in a clinical setting, such variability is to be expected, and assessment of the efficacy of treatment under such circumstances is preferable. Thirdly, the treatment time-period was limited to 12 weeks and the observation period to 20 weeks, potentially not allowing sufficient time for complete disease resolution in some of the Advocate® group dogs.

## Conclusions

Weekly, topical Advocate® therapy should be considered an alternative treatment for doramectin in dogs with benign esophageal spirocercosis, especially if clinical signs at the time of diagnosis are not severe, and in cases of avermectin susceptibility. However, Advocate® cannot be considered as effective as doramectin at the doses and treatment duration used herein, and higher Advocate® doses or longer treatment duration may be considered to achieve a quicker or more complete response.

## Abbreviations

CBC: Complete blood count
HRM: High-resolution melt analysis
HUVTH: Hebrew University Veterinary Teaching Hospital
PCR: Polymerase chain reaction
SC: Sub cutaneous
